# Sex-Based Anatomical Variations and Complication Risks in Pediatric Both-Bone Forearm Fractures: A Level of Evidence IV Retrospective Analysis

**DOI:** 10.3390/children12101404

**Published:** 2025-10-17

**Authors:** Onur Cetin, Ali Can Koluman, Mesut Demirkoparan, Ali Yucesan, Gokhan Karahan, Erhan Coskunol

**Affiliations:** 1 Department of Orthopedics and Traumatology, Istanbul Medipol University, 34815 Istanbul, Türkiye; drocetin@gmail.com; 2 Department of Orthopedics and Traumatology, Bakirkoy Dr. Sadi Konuk Training and Research Hospital, 34147 Istanbul, Türkiye; 3 Department of Orthopedics and Traumatology, Menderes State Hospital, 35471 Izmir, Türkiye; mesutdkoparan35@gmail.com; 4 Department of Orthopedics and Traumatology, Pine and Sakura City Hospital, 34490 Istanbul, Türkiye; aaliyucesan@gmail.com; 5 Department of Orthopedics and Traumatology, Academic Hospital,34664 Istanbul, Türkiye; hgk139@hotmail.com; 6 Department of Orthopedics and Traumatology, Ege University, 35100 Izmir, Türkiye; ecoskunol@hotmail.com

**Keywords:** forearm fracture, adolescents, conservative treatments, sex differences

## Abstract

**Highlights:**

**What are the main findings?**

- The study demonstrated that boys with both-bone forearm fractures had greater initial radius angulation and longer forearm bones compared to girls. Surgical intervention was required only in boys, whereas overall complication rates did not differ significantly between sexes.

**What is the implication of the main finding?**

- These results indicate that male-specific anatomical characteristics predispose boys to unstable fracture patterns and a higher likelihood of surgical treatment. Considering sex-based anatomical differences in treatment planning may improve early risk stratification and guide timely surgical decisions.

**Abstract:**

Background: Both-bone forearm fractures (BBFF) are among the most common pediatric injuries. While most cases in younger children can be managed non-operatively, older children and adolescents exhibit less predictable remodeling and longer healing times, potentially leading to higher complication rates. This study aimed to evaluate sex-based anatomical differences in BBFF and their association with complications. Methods: We retrospectively reviewed 163 patients (129 boys, 34 girls; age range: boys > 10 years, girls > 8 years, both < 16 years) with unilateral BBFF treated between 2017 and 2020. All underwent biplanar radiographs of both forearms pre-reduction, post-reduction, and at 8-week follow-up. Measurements included radius and ulna angulation, bone length, maximum radial bow (%), and location of maximum bow (mm). Complications and surgical interventions were recorded. Results: Boys demonstrated significantly greater initial radius angulation on the lateral view (*p* < 0.05) and longer radius and ulna lengths on the unaffected side (*p* < 0.05). Maximum radial bow (%) did not differ between sexes; however, the location of maximum bow varied between unaffected and fractured sides within each sex (*p* < 0.05). Twenty boys (15.5%) required surgery, compared with none of the girls (*p* = 0.007). Overall complication rates were 44.8% (*n* = 73) with no significant sex difference (*p* = 0.074). Conclusions: In older children and adolescents with BBFF, boys exhibit anatomical characteristics—such as longer forearms and greater initial angulation—associated with unstable fracture patterns and higher surgical intervention rates. Recognizing these differences may improve early risk stratification and management strategies. Level of Evidence: IV.

## 1. Introduction

Both-bone forearm fractures (BBFF) are among the most frequent injuries in the pediatric population, accounting for approximately 5–10% of all fractures in children [1,2]. By the age of 16, the cumulative fracture incidence is reported as 42% in boys and 27% in girls, with forearm fractures representing nearly 37% of all pediatric fractures [3,4]. Most BBFF cases in children result from indirect trauma, typically a fall on an outstretched hand with a rotational component [5].

In younger children (≤8–10 years), the forearm demonstrates a remarkable potential for healing and remodeling, allowing most fractures to be successfully managed non-operatively with cast immobilization for 4–6 weeks followed by gradual mobilization [6,7]. However, in older children and adolescents, remodeling capacity becomes less predictable, healing time is prolonged, and the risk of complications increases [8,9]. For this reason, surgical intervention is generally considered when residual angulation exceeds 10° after closed reduction in girls older than 8 years and boys older than 10 years with at least 2 years of growth remaining [10].

An important yet underexplored aspect of pediatric BBFF is the apparent male predominance [11,12]. While behavioral factors such as higher engagement in risk-prone activities may contribute to this disparity, anatomical differences between sexes could also influence fracture patterns, stability after reduction, and complication rates. Previous studies have rarely integrated radiographic anatomical measurements—such as radial bow characteristics and forearm length—into the analysis of sex-based outcomes in BBFF [11].

The present study aims to investigate sex-based anatomical variations in pediatric BBFF and their association with complication rates in older children and adolescents sustaining fractures from low-energy mechanisms such as a fall on an outstretched hand. We hypothesize that boys, due to their anatomical characteristics, present with more severely displaced fractures and consequently experience higher rates of fracture-related complications and surgical interventions compared to girls.

## 2. Materials and Methods

This retrospective observational study (Level of Evidence IV) was conducted at a single tertiary referral center with a high volume of pediatric trauma cases. All data was collected from electronic medical records and radiographic archives between January 2017 and September 2020. Ethical approval was obtained from the institutional review board (E.191678, 11 June 2021), and the study was performed in accordance with the Declaration of Helsinki, and parental informed consent was obtained for all participants.

**Figure 1:**
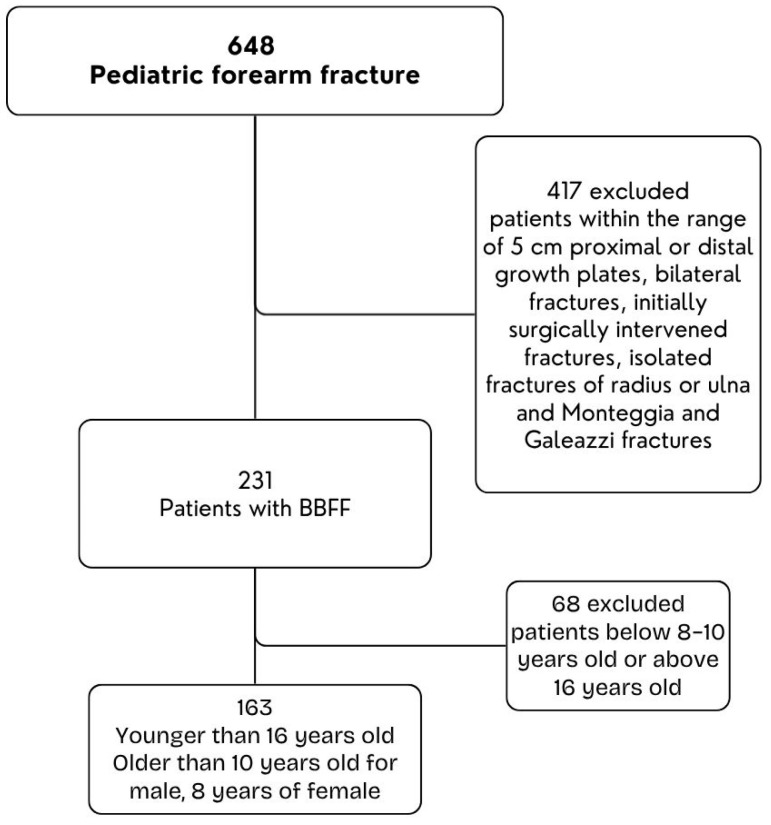
Flow Diagram of Patients.

From 648 pediatric patients diagnosed with BBFF between January 2017 and September 2020, we included those with unilateral fractures in boys > 10 years and girls > 8 years but <16 years of age, sustaining low-energy trauma and having complete radiographic follow-up for at least 8 weeks. Patients were excluded if they had bilateral fractures, fractures within 5 cm of either growth plate, initial surgical fixation before presentation, isolated fractures of the radius or ulna, Monteggia or Galeazzi fracture-dislocations, high-energy trauma, or underlying systemic/metabolic bone disorders. After applying the criteria, 163 patients were included: 129 boys (79.1%) and 34 girls (20.9%) (Figure 1).

## 3. Fracture Management

All fractures were initially managed in the emergency department under supervision of attending orthopedic surgeons, assisted by residents. Closed reduction was performed under appropriate analgesia/sedation, followed by application of a long-arm plaster cast with the elbow at 90° flexion and forearm in neutral rotation. Acceptable alignment was defined as ≤10° angulation and presence of bayonet apposition if necessary.

Standardized casting quality was assessed using the cast index and three-point index, as previously described [13]. Radiographs (anteroposterior and lateral) of both forearms were obtained before reduction on the fractured side, immediately after reduction, and again at the 8-week follow-up for both fractured and unaffected sides. Weekly radiographic controls were performed during the first month, followed by biweekly imaging until 8 weeks.

## 4. Radiographic Measurements

**Figure 2:**
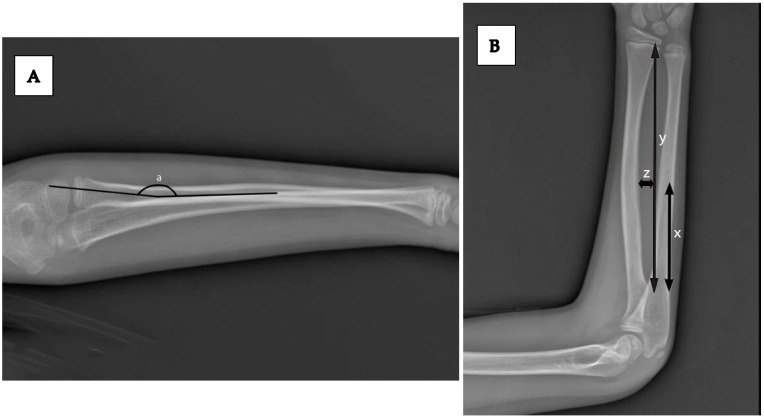
(**A**) Lateral forearm radiograph demonstrating measurement of the maximum radial bow (a), defined as the greatest perpendicular distance from the longitudinal axis of the radius to the point of maximal bowing. (**B**) Anteroposterior forearm radiograph illustrating measurement of the location of the maximum radial bow. The total radial length is represented as y. The distance from the bicipital tuberosity to the site of maximum bow is x. The maximum radial bow is expressed as z, the greatest distance from the longitudinal axis to the bow. The relative location of maximum bow is expressed as the ratio *x*/*y* (%).

All radiographic measurements were performed digitally using the hospital PACS system by two independent observers (one pediatric orthopedic surgeon, one senior resident), each blinded to patient sex and clinical outcomes. Measurements included the following:

- Angulation of radius and ulna in AP and lateral views
- Bone length (radius and ulna) on the unaffected side
- Maximum radial bow (%) and location of maximum bow (mm), as described by Schemitsch and Richards and modified by Firl and Wünsch [14,15]. (Figure 2).

Intra- and interobserver reliability were assessed in a random subset of 30 patients using the intraclass correlation coefficient (ICC).

## 5. Outcome Measures

Primary outcomes:

- Anatomical differences between sexes (angulation, bone length, bow characteristics)
- Complication rates (major: re-reduction failure, loss of reduction, need for surgery, pre-compartment syndrome; minor: swelling, mild post-reduction pain, tenderness)

Secondary outcomes:

- Surgical intervention rates
- Time to radiographic healing (defined as callus bridging the fracture gap in at least three cortices)

### Statistical Analysis

All statistical analyses were performed using IBM SPSS Statistics, version 26 (IBM Corp., Armonk, NY, USA). The distribution of continuous variables was examined with normality tests, distribution plots, and evaluation of sample sizes, and none met the assumptions of normal distribution. Continuous variables are therefore presented as medians with interquartile ranges (IQR), and group comparisons were performed using the Mann–Whitney U test. Categorical variables were analyzed using Pearson’s chi-square test when all expected cell counts were ≥5, and Fisher’s exact test when expected frequencies were <5. All statistical tests were two-tailed, and the significance level was set at α = 0.05. Effect sizes were calculated to improve clinical interpretation. Cohen’s d was reported for continuous outcomes and φ coefficients for categorical outcomes, together with their 95% confidence intervals (CIs). To account for potential confounding, multivariate regression analyses were performed. Linear regression models were applied for continuous outcomes (e.g., angulation, bone length), and logistic regression models for binary outcomes (e.g., complications, surgical intervention). Independent variables included in the models were sex, age, height, and weight. All regression models were checked for multicollinearity and goodness-of-fit. Adjusted odds ratios (OR) and mean differences with 95% CIs were reported. Finally, a post hoc power analysis was performed to evaluate the adequacy of the sample size. With 129 boys and 34 girls, the study had approximately 80% power to detect medium effect sizes (Cohen’s d ≈ 0.5, OR ≈ 2.0) at α = 0.05 (two-tailed), but limited power for detecting small effect sizes or rare outcomes such as complications. Because multiple anatomic variables were compared between sexes, we additionally controlled the false discovery rate (FDR) at q = 0.05 using the Benjamini–Hochberg procedure. Both raw and FDR-adjusted *p*-values are reported.

## 6. Results

A total of 163 patients met the inclusion criteria, comprising 129 boys (79.1%) and 34 girls (20.9%), with a mean age of 11.5 years (SD 1.6; range 8–15 years) (Table 1). Boys were significantly overrepresented in the study cohort (*p* < 0.05). The quality of casting was comparable between sexes, with identical median cast index values of 0.8 (IQR 0.7–0.9; *p* = 0.443) and three-point index values of 0.5 (IQR 0.5–0.6; *p* = 0.895), indicating no significant difference in immobilization technique.

Intraobserver ICC values ranged from 0.87 to 0.93, and interobserver ICC values ranged from 0.82 to 0.90, confirming excellent reproducibility of the radiographic measurements.

Radiographic evaluation revealed no sex-related difference in initial radius angulation on the anteroposterior (AP) view or ulna angulation on either the AP or lateral views; however, boys exhibited significantly greater initial radius angulation on the lateral view (*p* < 0.05) (Table 2). On the unaffected side, both the radius and ulna were significantly longer in boys compared to girls (*p* < 0.05). The maximum radial bow (%) did not differ between sexes, but the location of the maximum radial bow (mm) showed a significant difference between unaffected and fractured sides within each sex (*p* < 0.05). Reference values reported by Firl and Wünsch (maximum radial bow 7.21 ± 1.03 mm; location 60.39 ± 3.74%) were used as a benchmark for interpretation [15].

During follow-up, twenty boys (15.5% of male patients) required surgical intervention, whereas no girl underwent operative treatment (*p* = 0.007) (Table 3). Overall, 73 patients (44.8%) experienced at least one complication, but the total complication rate did not differ significantly between sexes (*p* = 0.074). Among major complications, 38 patients (23.3%) presented with insufficient initial reduction, all of whom underwent re-reduction; however, in 10 boys, alignment remained unacceptable, necessitating surgery. Loss of reduction during casting occurred in 27 patients (16.6%), including 22 boys and 5 girls; all underwent repeat closed reduction, but 5 boys subsequently required surgical fixation. Three boys sustained refractures after cast removal, all treated operatively. Impending compartment syndrome developed in 8 patients during the casting period; in 2 boys, cast removal and urgent surgical intervention were performed to prevent progression. Minor complications were more common but self-limiting, including swelling (n = 69), mild pain following reduction (n = 33), and localized tenderness (n = 29). No neurological deficits were observed in any patient.

In multivariate linear regression, male sex remained a significant independent predictor of radius angulation on the lateral view (β = +6.2°, *p* = 0.003) and longer radius and ulna lengths (β = +17.4 mm and +15.9 mm, both *p* < 0.001), even after adjustment for age, height, and weight (Table 4). Logistic regression confirmed that boys had higher odds of requiring surgical intervention compared with girls (adjusted OR = 3.1, 95% CI: 1.2–8.4, *p* = 0.022). However, sex was not independently associated with overall complication rates (adjusted OR = 1.5, 95% CI: 0.7–3.3, *p* = 0.284) (Table 5). These findings indicate that the anatomical differences and higher surgical intervention rates in boys are not fully explained by age or growth-related parameters.

## 7. Discussion

This study examined sex-based anatomical differences in pediatric both-bone forearm fractures (BBFF) and their association with outcomes including complication rates, re-displacement, and surgical intervention. Although overall complication rates did not differ significantly between sexes, boys showed greater initial radius angulation in the lateral view, longer radius and ulna lengths, and a significantly higher rate of surgical treatment. These anatomical characteristics observed in boys may be associated with a higher likelihood of unstable fracture patterns and less favorable outcomes after closed reduction, even when the mechanism of injury is similar.

Our regression analyses, adjusted for age, height, and weight, reinforce that male sex remains an independent predictor of increased initial lateral radius angulation, longer forearm bone lengths, and the likelihood of operative intervention. However, it is essential to interpret these results as associations only; the retrospective design limits causal inferences. Anatomical explanations remain plausible mechanisms rather than definitively proven causes.

The male predominance observed in our cohort (79.1%) is consistent with prior epidemiological data reporting a 63–80% male-to-female ratio in pediatric BBFF [12]. While behavioral factors, such as higher engagement in risk-prone activities and earlier return to sports, may partly explain this disparity, our data suggest that intrinsic anatomical differences—particularly longer forearm bones and altered radial bow location—may also contribute. Ryan et al. reported no sex-based differences in weight status, seasonal distribution, or trauma severity [12], supporting the hypothesis that structural factors, rather than solely behavior, may underlie the higher incidence and more complex presentations observed in boys.

Most pediatric BBFF cases can be managed non-operatively with excellent functional recovery, unlike in adults. However, older children and adolescents have reduced remodeling capacity and longer healing times, which increase the risk of loss of reduction and need for secondary surgical fixation [8,9]. In the literature, surgical intervention rates following failed conservative management range from 5.6% to 31.6% [16,17,18,19,20]. In our series, 15.5% of boys required surgery, compared with none of the girls, despite comparable casting quality. This aligns with previous reports that inadequate initial reduction and subsequent re-displacement are the most frequent causes for conversion to surgery [21,22,23]. The exclusive occurrence of refractures in boys in our cohort further suggests a potential sex-linked biomechanical susceptibility.

Initial reduction failure and subsequent re-displacement occurred more frequently in boys in our series, and both scenarios often necessitated operative intervention. The exclusive occurrence of refractures in boys further supports the possibility of sex-linked biomechanical susceptibility. Several studies have reported that malalignment after reduction occurs in 18–51% of conservatively treated BBFF [13,21,24]. While some authors argue that angulation alone is a poor predictor of long-term function [25,26], others have shown that initial angulation beyond 15–20° is associated with increased complication risks and poorer outcomes [25,27,28,29,30]. In our cohort, boys more frequently presented with initial lateral radius angulation above 20°, a threshold previously linked to functional and cosmetic deficits [25,27,28,29,30]. Even though reduction corrected angulation to within acceptable limits in both sexes, the initial severity in boys may explain their higher rate of unstable outcomes and surgical conversion.

Recent evidence further supports the influence of anatomical and treatment-related factors on long-term stability. Husum et al. reported that in diaphyseal forearm fractures, nonoperative treatment carried nearly a tenfold higher risk of refracture compared with flexible intramedullary nailing, while closed reduction doubled the risk of malunion compared to intramedullary fixation [31]. These findings are consistent with our observation that anatomical severity, more common in boys, predisposes to poorer outcomes despite adequate casting. Similarly, Wang et al. demonstrated in a multicenter series that adding ulna fixation to percutaneous pinning in distal both-bone fractures did not improve functional outcomes, suggesting that surgical indications should be individualized based on anatomical severity rather than uniform protocols [32]. Furthermore, a recent meta-analysis concluded that while both intramedullary and plate fixation yield high union rates in skeletally immature patients, plate fixation better restores radial bow in older children and adolescents [33]. This highlights the clinical relevance of our findings: anatomical differences such as increased angulation and longer bone length in boys may challenge the maintenance of alignment with conservative treatment alone.

From a clinical perspective, these results suggest that boys who present with significant lateral radius angulation (for example above 20°), or those whose forearm bone lengths exceed expected norms for their age, may warrant more cautious management: closer radiographic monitoring, consideration of earlier surgical fixation if reduction is unstable, or stricter criteria for acceptable post-reduction alignment.

This study has several limitations. First, its retrospective design introduces potential selection bias, and confounding variables such as detailed activity levels, handedness, and specific sports participation were not consistently available. Second, the study was conducted in a single tertiary referral center, which may limit generalizability. Third, and most importantly, the sample size was highly imbalanced (129 boys vs. 34 girls). While post hoc power analysis indicated adequate power to detect medium effect sizes, the study remained underpowered to detect small differences or rare outcomes such as complications. This imbalance reduces the strength of sex-based comparisons and warrants cautious interpretation of negative findings. Moreover, while some anatomical explanations are suggested, they remain speculative without biomechanical or longitudinal functional validation. Future research should include prospective multicenter cohorts with more balanced sex distributions, inclusion of skeletal maturity (e.g., Risser grade, bone age), assessment of bone geometry (including radial bow magnitude and offset), and long-term functional outcomes (range of motion, pronation/supination, patient-reported measures). Investigations into biomechanical modelling or finite element analysis may help determine whether observed anatomical differences translate into meaningful differences in mechanical stability.

## 8. Conclusions

In older children and adolescents with both-bone forearm fractures, boys more frequently present with anatomical characteristics—such as longer forearm bones and greater initial radius angulation—that may be associated with unstable fracture patterns and a higher likelihood of surgical intervention. Although overall complication rates were similar between sexes, the occurrence of operative cases exclusively in boys highlights the potential clinical relevance of these anatomical differences. Incorporating sex-specific anatomical considerations into initial evaluation may improve risk stratification and support timely treatment decisions. Future multicenter prospective studies with balanced cohorts are needed to validate these associations.

## Figures and Tables

**Table 1:** Demographic Characteristics of Patients with Both-Bone Forearm Fractures.

		N (%)	95% CI
Sex	Male	129 (79.1%)	72.2–84.5%
	Female	34 (20.9%)	15.5–27.8%
Age _(Mean, ±SD)_		11.5 (±1.6)	11.0–12.0%
Side	Left	101 (62.0%)	54.4–69.0%
	Right	62 (39.0%)	31.0–45.6%

**Table 2:** Radiological evaluations of pre- and post-reduction results and comparison with contralateral extremity.

		Sex				
		Male	Female			
		Median (IQR _(25; 75)_ ***) [95% CI]	Median (IQR _(25; 75)_) [95% CI]	*p* * Raw Value	Adjusted *p* † (BH-FDR)	D ** Cohen
Radius Angulation on AP View	Initial	9.8 (4.2; 17.1) [8.6–11.7]	8.4 (1.7; 13.1) [5.4–12.3]	0.314	0.48	0.177
	Post-reduction	2.0 (1.1; 4.3) [1.8–2.8]	2.2 (0.9; 4.1) [1.5–2.4]	0.962	0.96	0.008
	Change	7.4 (0.4; 13.2) [7.0–8.1]	3.3 (0.3; 12.8) [2.8–3.5]	0.637	0.79	0.083
Radius Angulation on Lateral View	Initial	26.3 (18.4; 31.5) [23.6–27.6]	18.5 (13; 24.8) [13.8–21.9]	**0.010**	**0.03**	0.459
	Post-reduction	4.3 (1.7; 8.0) [3.5–5.4]	5.4 (2.9; 8.7) [2.5–6.7]	0.569	0.79	0.100
	Change	21 (11.0; 27.2) [18.1–26.2]	11.8 (8.7; 16.4) [9.9–13.5]	**0.017**	**0.04**	0.426
Ulna Angulation on Ap View	Initial	10.1 (5.5; 19.1) [9.1–13.1]	8.7 (1.6; 26.4) [7.9–13.7]	0.735	0.88	0.059
	Post-reduction	3.1 (1.5; 5.4) [1.9–3.4]	3.0 (1.7; 5.5) [1.3–4.7]	0.960	0.96	0.009
	Change	6.5 (1.3; 15.7) [4.9–8.2]	6.8 (−0.2; 21.8) [5.1–8.8]	0.931	0.96	0.015
Ulna Angulation on Lateral View	Initial	25.0 (13.3; 35.4) [19.2–26.7]	18.7 (9.5; 26.0) [11.0–23.6]	0.100	0.20	0.291
	Post-reduction	3.6 (1.6; 6.8) [2.7–4.1]	1.6 (0.7; 6.7) [1.2–2.0]	0.142	0.24	0.259
	Change	20 (9.6; 31.6) [14.9–23.2]	17.8 (8.2; 22.7) [15.0–21.2]	0.244	0.39	0.204
Lenght of Radius (mm)	Unaffected side	206.8 (197; 222) [203.5–216.7]	187.1 (184; 203) [184.0–202.8]	**<0.001**	**<0.001**	0.798
	Fractured side (Final X-Ray)	210.3 (198; 223) [205.8–219.4]	190.9 (186; 210) [187.8–209.8]	**0.001**	**0.003**	0.672
Lenght of Ulna (mm)	Unaffected side	214.6 (204; 234) [211.5–229.0]	194.6 (190; 214) [192.2–213.8]	**0.001**	**0.003**	0.725
	Fractured side (Final X-Ray)	217.5 (206; 236) [211.6–230.0]	205.0 (195; 221) [198.3–221.2]	**0.008**	**0.02**	0.551
Location of Maximum Radial Bowing (mm)	Unaffected side	62.0 (60; 65) [61.0–64.0]	65.0 (62; 69) [63.0–68.0]	**0.010**	**0.03**	0.504
	Fractured side (Final X-Ray)	64.0 (59; 67) [62.0–65.0]	63.0 (59; 65) [62.0–63.5]	0.671	0.79	0.081
Maximum Radial Bow (%)	Unaffected side	7.5 (6.8; 8.1) [7.1–7.8]	6.9 (6.7; 7.8) [6.7–7.0]	0.062	0.12	0.364
	Fractured side (Final X-Ray)	7.1 (7.0; 7.7) [6.9–7.6]	7.0 (6.9; 7.8) [6.7–7.7]	0.597	0.79	0.101

* Mann–Whitney U test was used for comparisons between sexes. ** Effect size (Cohen’s d) values are presented with 95% confidence intervals. Cohen’s d interpretation: 0.0–0.1 no effect, 0.2–0.4 small effect, 0.5–0.7 intermediate effect, ≥0.8 large effect. *** IQR: Interquartile range (25th–75th percentiles). † False-discovery-rate–adjusted *p* using Benjamini–Hochberg (q = 0.05). Significant after adjustment shown in bold.

**Table 3:** Complication rates, reduction quality, re-displacement and operation rates between sexes.

		Sex					
		Male		Female			
		N	N %	N	N %	*p* * _Value_	φ ** _(Eff. Size)_
Major Complications	Yes	63	48.8	10	29.4	0.074	0.15
	No	66	51.2	24	70.6		
Closed Reduction	Unacceptable	34	26.4%	4	11.8%	0.118^ ‡^	0.12
	Acceptable	95	73.6%	30	88.2%		
Re-displacement	Yes	22	17.0%	5	14.7%	0.783 ^‡^	0.0170
	No	107	83.0%	29	85.3%		
Operation	Yes	20	19.2%	0	0.0%	**0.007** ^ ‡^	0.2
	No	84	80.8%	30	100.0%		

* Categorical variables were analyzed using Pearson’s chi-square test and Fisher’s exact test. ^‡^ Fisher’s exact test was applied when expected cell counts were <5 (closed reduction quality, re-displacement, surgical intervention). ** Effect sizes (φ) are provided with 95% confidence intervals.

**Table 4:** Multivariate linear regression analysis for continuous radiographic outcomes.

Outcome	Independent Predictor	β (Coefficient)	95% CI	*p* Value
Radius Angulation on AP View	Male Sex (Ref = Female)	+6.2	2.1–10.3	0.003
	Age	0.8	−0.2–1.8	0.115
	Height	0.05	−0.03–0.13	0.21
	Weight	0.3	−0.5–1.1	0.445
Lenght of Radius	Male Sex (Ref = Female)	+17.4	10.8–23.9	<0.001
	Age	+1.2	0.2–2.3	0.018
	Height	+0.11	0.05–0.17	<0.001
	Weight	+0.6	–0.3–1.5	0.180
Lenght of Ulna	Male Sex (Ref = Female)	+15.9	9.2–22.6	<0.001
	Age	+1.0	–0.1–2.0	0.071
	Height	+0.09	0.02–0.16	0.009
	Weight	+0.4	–0.5–1.3	0.374

**Table 5:** Multivariate logistic regression analysis for binary outcomes.

Any Complication (Yes)	Independent Predictor	Adjusted OR	95% CI	*p* Value
Surgical Intervention (Yes)	Male Sex (Ref = Female)	3.1	1.2–8.4	0.022
	Age	1.1	0.8–1.5	0.490
	Height	1.01	0.98–1.03	0.390
	Weight	0.9	0.6–1.4	0.670
Complication (Yes)	Male Sex (Ref = Female)	1.5	0.7–3.3	0.284
	Age	1.0	0.8–1.2	0.880
	Height	1.0	0.98–1.02	0.765
	Weight	1.1	0.9–1.4	0.320

## Data Availability

The data supporting the findings of this study are available from the corresponding author upon reasonable request.

## Abbreviations

BBFF: Both-Bone Forearm Fractures
